# Global Analysis of Mannitol 2-Dehydrogenase in *Lactobacillus reuteri* CRL 1101 during Mannitol Production through Enzymatic, Genetic and Proteomic Approaches

**DOI:** 10.1371/journal.pone.0169441

**Published:** 2017-01-06

**Authors:** Maria Eugenia Ortiz, Juliana Bleckwedel, Silvina Fadda, Gianluca Picariello, Elvira M. Hebert, Raúl R. Raya, Fernanda Mozzi

**Affiliations:** 1 Centro de Referencia para Lactobacilos (CERELA)-CONICET, Chacabuco 145, San Miguel de Tucumán, Tucumán, Argentina; 2 Istituto di Scienze dell' Alimentazione–CNR, Avellino, Avellino, Italy; University of Torino, ITALY

## Abstract

Several plants, fungi, algae, and certain bacteria produce mannitol, a polyol derived from fructose. Mannitol has multiple industrial applications in the food, pharmaceutical, and medical industries, being mainly used as a non-metabolizable sweetener in foods. Many heterofermentative lactic acid bacteria synthesize mannitol when an alternative electron acceptor such as fructose is present in the medium. In previous work, we reported the ability of *Lactobacillus reuteri* CRL 1101 to efficiently produce mannitol from sugarcane molasses as carbon source at constant pH of 5.0; the activity of the enzyme mannitol 2-dehydrogenase (MDH) responsible for the fructose conversion into mannitol being highest during the log cell growth phase. Here, a detailed assessment of the MDH activity and relative expression of the *mdh* gene during the growth of *L*. *reuteri* CRL 1101 in the presence of fructose is presented. It was observed that MDH was markedly induced by the presence of fructose. A direct correlation between the maximum MDH enzyme activity and a high level of *mdh* transcript expression during the log-phase of cells grown in a fructose-containing chemically defined medium was detected. Furthermore, two proteomic approaches (2DE and shotgun proteomics) applied in this study confirmed the inducible expression of MDH in *L*. *reuteri*. A global study of the effect of fructose on activity, *mdh* gene, and protein expressions of MDH in *L*. *reuteri* is thus for the first time presented. This work represents a deep insight into the polyol formation by a *Lactobacillus* strain with biotechnological potential in the nutraceutics and pharmaceutical areas.

## Introduction

Mannitol, an alditol derived from fructose, is widely distributed in nature and is the most abundant polyol in the plant kingdom. Furthermore, it is produced by a large number of filamentous fungi of the *Aspergillus* and *Penicillium* genera, by yeasts belonging to the *Candida* genus, and by bacteria such as *Pseudomonas putida* and heterofermentative lactic acid bacteria (LAB) [1–5].

Mannitol has been classified as a GRAS (*Generally Recognized as Safe*) compound by the Food and Drug Administration (FDA) [6] and as a food additive (E421) by the European Union, being considered a safe food ingredient [7]. Mannitol is used in the food industry as a sweetener (60% sweetness compared to sucrose), especially in products for diabetic patients [6,8]. In addition, it is appreciated for its antioxidant and non-cariogenic effects as it is poorly metabolized by the oral microbiota [9,10]. Because of this property along with its ability to provide a cooling sensation in the mouth (positive enthalpy of dissolution, 120.9 kJ/kg) [2] and its low hygroscopicity, mannitol has been employed to sprinkle the surface of "sugar-free" chewing gums [11], to reduce moisture absorption, and to increase the fluidity in powdered instant desserts [4]. In addition, it has been used as a texturizing and anticaking agent [12]. In medicine, mannitol is applied as a potent osmotic diuretic while in the pharmaceutical industry it is used to mask undesirable flavors [13].

In previous work we demonstrated that the heterofermentative strain *Lactobacillus reuteri* CRL 1101 efficiently produced mannitol in both rich and simplified culture media containing sugarcane molasses as carbon source [14,15]. Maximum mannitol concentrations (38 and 41.5 g/L) and yields (Y_Mtl:_ 86.9 and 105%) were attained using 7.5% (w/v) of sugar from sugarcane molasses when grown in agitated cultures at 37°C under free- and constant (5.0)-pH conditions, respectively, after 24 h of incubation.

**Fig 1:**
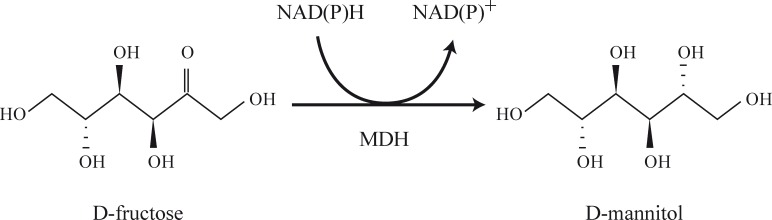
Conversion of fructose into mannitol catalyzed by the mannitol 2-dehydrogenase (MDH) enzyme.

Mannitol 2-dehydrogenase (MDH), the enzyme responsible for the one-step conversion of fructose into mannitol (Fig 1), requires either NADH or NADPH as cofactors. While NADH-dependent MDH enzyme (EC 1.1.1.67) was first isolated from *Lactobacillus brevis* [16] and purified from strains of *Leuconostoc mesenteroides* [17,18], *Torulaspora delbrueckii* [19], *Rhodobacter sphaeroides* [20], *Pseudomonas fluorescens* [21], and the red algae *Caloglossa leprieurii* [22], the NADPH-dependent MDH (EC 1.1.1.138) was isolated and purified from *Aspergillus parasiticus* [23], *Zymomonas mobilis* [24], *Gluconobacter suboxydans* [25], and from several *Lactobacillus* strains including *Lactobacillus sanfranciscensis* [26], *Lactobacillus intermedius* [27], and *L*. *reuteri* [28].

Although the MDH activity has been evaluated in intracellular extracts from several LAB species such as *L*. *intermedius* [27], *L*. *sanfranciscensis* [26], and *Leuc*. *mesenteroides* [29], no studies on the *mdh* gene expression have been performed in any LAB. In this work, the MDH activity in intracellular extracts of *L*. *reuteri* CRL 1101 together with the effect of the presence of fructose, the precursor sugar for mannitol biosynthesis, on the *mdh* gene and protein expression were evaluated. Its relative transcript levels were quantified by reverse transcription-coupled quantitative PCR (qPCR) technique. In addition, the enzymatic and/or metabolic shifts in *L*. *reuteri* CRL 1101 affected by the presence of the alternative electron acceptor fructose were investigated using both the “classical” two dimensional electrophoresis (2DE) and gel-free shotgun proteomics approaches.

## Materials and Methods

### Bacterial growth conditions and culture medium

*L*. *reuteri* CRL 1101 belongs to the Culture Collection of CERELA, San Miguel de Tucumán, Argentina. The strain was grown in MRS broth or in a Chemically Defined Medium (CDM) with 2% (w/v) glucose and 5% (w/v) fructose (MRS_GF_ and CDM_GF_, respectively) as carbon sources at 37°C for 24 h. Glucose was added to promote cell growth and fructose was needed for mannitol production. MRS and CDM with 7% (w/v) glucose (MRS_G_ and CDM_G_) were used as controls. CDM was prepared according to Hébert et al. [30] with the following modifications: i) glucose concentration of the stock solution was changed from 200 to 400 g/L to give a final glucose concentration of 20 or 70 g/L as appropriate; ii) fructose was added to the medium when needed (50 g/L, final concentration); iii) FeSO_4_.7H_2_O and inosine were omitted as they were not essential for growth of *L*. *reuteri* CRL 1101; and iv) the MnSO_4_.H_2_O concentration of the stock solution was doubled from 2.5 to 5.0 g/L as required for optimal cell growth. The culture medium was always prepared immediately before use. Cell growth was determined periodically by measurement of the optical density at 560 nm (OD_560_) and cell count (CFU/mL) by plating diluted samples in physiological solution (0.85% NaCl, w/v) in MRS agar (MRS plus 12 g agar/L). CDM cultures of *L*. *reuteri* CRL 1101 for qPCR or 2DE assays (see below) were done in duplicate of at least two independent experiments for each growth condition (presence and absence of fructose at 8 and 24 h of incubation).

### Determination of mannitol and carbohydrates by High Performance Liquid Chromatography (HPLC)

Samples withdrawn from different microbial growth time points (0, 4, 8, and 24 h) were centrifuged (8,000×*g*, 10 min, 4°C) and the resulting supernatants were deproteinized with Carrez A and B reagents [14]. Mannitol, glucose, and fructose concentrations were determined by HPLC using an Aminex HPX-87P column (Bio-Rad Laboratories Inc.; San Francisco, CA, USA) at 85°C using Milli-Q water as mobile phase [14].All components were analyzed by HPLC [pump Smartline 100, refractive index (RI) detector K-2301, Smartline autosampler 3800Plus, Knauer, Berlin, Germany with a Zeltec ZC90 oven, Buenos Aires, Argentina]. The elution rate was 0.6 mL/min. All data were analyzed using the Eurochrom Basic Edition for Windows software.

### Preparation of intracellular protein extracts

Cells were harvested by centrifugation (8,000×*g*, 10 min, 4°C) using 30 mL of fermented broth from different incubation times (4, 8, and 24 h) and washed three times with cold 50-mM potassium phosphate buffer (pH 5.5). The wet pellets were mixed with glass beads (150–212 μm diameter, Sigma-Aldrich Co., St. Louis, MO, USA) and further re-suspended in the same buffer in a 1:2:1 (cell:buffer:bead) ratio. Then, cells were disrupted using a Mini-BeadBeater-8 cell disrupter (Biospec Products Inc., Bartlesville, OK, USA) at maximum speed for 10 min (5 cycles of 2 min each, with 2-min intervals on ice among cycles). To remove cell debris, unbroken cells, and glass beads samples were centrifuged (14,500×*g*, 5 min, 4°C), and the supernatants were immediately used for enzyme assays. For proteomic assays (2DE and Shotgun), the supernatants were treated with phenylmethylsulfonyl fluoride (PMSF, 1 mM) to inhibit serine-proteases. The total protein concentrations of the protein extracts were determined by the Bradford method (Bio-Rad Laboratories Inc., Hercules, CA, USA) following the manufacturer's instructions and using bovine serum albumin (BSA, 0.05–0.50 mg/mL) as standard. Protein extracts were lyophilized for further analysis.

### Determination of MDH activity

Enzymatic activity was determined according to the method described previously [15]. Briefly, protein extracts were diluted to obtain a protein concentration of 0.5 mg prot/mL. The reaction mixtures of enzyme assays contained 50 μL of 200 mM sodium phosphate buffer (pH 5.5), 50 μL of 2 mM NADPH (Sigma-Aldrich Chemical Co.), 50 μL of Milli-Q water, and 10 μL of diluted protein extract. The mixture was incubated at 37°C for 2 min, and the reaction was started by the addition of 40 μL of 1 M fructose (Sigma-Aldrich Chemical Co.). The disappearance of NADPH was spectrophotometrically monitored by measuring the absorbance at 340 nm (ε_340_: 6,220/M cm) for 5 min. NADPH was used as cofactor as the MDH enzyme showed higher affinity for this cofactor rather than for NADH [14]. One unit (U) of MDH activity was defined as the amount of enzyme required to catalyze the disappearance (fructose reducing direction) of 1 μmol of NADPH per minute under the experimental conditions used. Specific MDH activity was expressed in units per milligram of protein. All enzymatic assays were done in duplicate of three independent experiments. Statistical data analysis was performed with GraphPad Prism 6.0 (GraphPad Software, Inc., San Diego, CA) using two-way analysis of variance (ANOVA) analyzing the effect of the presence of fructose and the period of incubation, followed by the Bonferroni post-test. A value of *p<*0.05 was considered statistically significant.

### qPCR for relative quantification of the *mdh* gene expression

Total RNA was isolated from cell pellets containing approximately 1×10^8^ CFU/mL from 8 h- (mid-exponential growth phase) and 24 h- (stationary growth phase) incubation samples. RNA isolation was performed using the NucleoSpin^®^ RNA II kit (Macherey-Nagel GmbH & Co. KG; Düren, Germany) according to the manufacturer’s instructions with the following modifications: pellets were re-suspended in 100 μL of TE buffer (10 mM Tris-HCl, 1 mM EDTA, pH 8) containing 2 mg/mL of lysozyme (Sigma-Aldrich Co.) and were incubated for 2 h at 37°C. Then, 350 μL of RA1 buffer (provided by the kit), 3.5 mL of β-mercaptoethanol, and glass beads (diameter 150–212 μm, Sigma-Aldrich Co.) in a 1:1 (cell:bead) ratio were added to each sample. Subsequently, cells were disrupted with a Mini-BeadBeater-8 cell disrupter (BioSpec Products Inc.) at maximum speed with 5 cycles of 2 min each cycle, with intervals of 2 min on ice among cycles. To remove glass beads and cell debris, samples were centrifuged (5,000×*g*, 3 min, 4°C) and the protocol described by the manufacturer was followed. Purified RNA was re-suspended in 60 μL of ultrapure DNase/RNase-free distilled water. RNA precipitation was done with absolute ethanol and 3M sodium acetate (pH 5.4) at -20°C overnight. Samples were centrifuged (13,000×*g*, 5 min, 4°C) and re-suspended in 30 μL (corresponding to the half of the original amount) of ultrapure DNase/RNase-free distilled water. The absence of residual DNA was confirmed by conventional PCR using the purified RNA as template. RNA samples were quantified with a Qubit^®^ 2.0 fluorometer (Invitrogen^TM^, Life Technologies Co., Carlsbad, CA, USA) using Qubit^®^ HS RNA Assay Kit (Molecular Probes^TM^, Life Technologies Co.) and stored at -70°C.

The synthesis of cDNA was performed using reverse transcription in a My Cycler^TM^ Thermal Cycler System with Gradient Option (Bio-Rad Laboratories Inc.) using ~0.5 μg of total RNA and SuperScript III First-Strand Synthesis SuperMix kit (Invitrogen^TM^, Life Technologies Co.) according to the manufacturer's instructions. The cDNA synthesis was confirmed by conventional PCR. The cDNA obtained was quantified using Qubit^®^ ssDNA Assay Kit (Invitrogen^TM^, Life Technologies Co.) and stored in aliquots at -70°C until use.

Specific primers pairs were designed using the PrimerQuest tool (Integrated DNA Technologies Inc.) available online, to amplify the *mdh* gene as well as four genes commonly used as normalizing (housekeeping) genes: *16S rRNA*, *gyrB*, *pyrG*, and *leuS*. All primers were designed to obtain fragments of 100 to 200 bp size based on the annotated genome of *L*. *reuteri* DSM 20016 (http://www.ncbi.nlm.nih.gov/nuccore/NC_009513.1). The primers were purchased from Sigma-Aldrich Co. The specificity of each primer pair and fragment sizes were verified before quantitative analysis by conventional PCR using genomic DNA (gDNA) as template and subsequent visualization by agarose gel electrophoresis. Then, a qPCR assay to check the constant expression of the housekeeping genes in presence and absence of fructose was performed. The *pyrG* gene was selected as normalizing gene since its expression remained constant under all the assayed conditions. The sequences of the primers used were: *mdh* forward primer 5'-AAC CGG AAG CAC TTT GGC GTT AAG-3' and *mdh* reverse primer 5'-GCA GCT GCA AGT GCT TGT TCT TGA-3'; *pyrG* forward primer 5'-ATC GTT GCT GCC TCT TTA GGA CGA-3' and *pyrG* reverse primer 5'-GGT CCA AAT CAG TTT CTG TGC CGT-3'.

All qPCR assays were performed on an iQ^TM^5 Multicolor Real-Time PCR Detection System iCycler (Bio-Rad Laboratories Inc.). Amplification products were detected by using iQ^TM^ SYBR^®^ Green Supermix (Bio-Rad Laboratories Inc.). Each reaction was performed in duplicate, containing 1X iQ^TM^ SYBR^®^ Green Supermix, 200 nM of each primer, and 30 ng of total cDNA. Positive controls with genomic DNA and no template controls (NTC, negative controls) were also included. The amplification program consisted of 1 cycle of 94°C for 5 min and 40 cycles of amplification (94°C for 1 min, 55°C for 1 min, and 72°C for 30 s), followed by a melting curve (81 cycles of 10 sec at 60°C). Two independent qPCR assays were performed for each condition (presence and absence of fructose, and 8 and 24 h of incubation). The relative expression of the *mdh* gene in different conditions was estimated according to the 2^-ΔΔCT^ method [31]. The condition of 8 h of incubation in the presence of glucose was used as control. Values reported are the fold changes between each condition and the control (given the value 1) and were normalized against the *pyrG* gene expression. Statistical data analysis was performed with GraphPad Prism 6.0 (GraphPad Software, Inc., San Diego, CA) using one sample *t*-test comparing all values with an hypothetical value of one (control condition). Differences between groups were considered to be significant at a *p* value of <0.05.

### Two-Dimensional Electrophoresis (2DE)

Intracellular protein extracts containing 600 μg protein were precipitated with 20% (w/v) TCA for 30 min on ice bath and then washed 3 times with -20°C cold acetone and centrifuged (12,000×*g*, 20 min, 4°C). 2DE was performed according to O'Farrell [32]. The precipitates were re-suspended in 340 μL of isoelectrofocusing (IEF) buffer containing 7M urea, 2M thiourea, 4% (w/v) 3-[(3-cholamidopropyl)-dimethylammonio]-1-propanesulfonate (CHAPS), 0.5% (v/v) ampholyte IPG (pH 4–7), 1% (w/v) DTT, and bromophenol blue. The samples were used to passively rehydrate the immobilized pH gradient (IPG) strips (Immobiline DryStrip Gels, pH 4–7, 18 cm, GE Healthcare; Uppsala, Sweden) at room temperature (RT) for 16 h. The IEF was performed using an EttanIPGphor 3 System (GE Healthcare) achieving 52,000 final V h. For the second dimension, IEF strips were equilibrated at RT in 6 M urea, 2% (w/v) SDS, 30% (w/v) glycerol, 50 mMTris-HCl, pH 8.0, containing alternatively 50 mM DTT (15 min) and then 400 mM iodoacetamide (15 min in the dark). SDS-PAGE was performed onhomogeneous12% (w/v) polyacrylamide gels at the constant current of 15 mA/gel at 15°C (approximately 16 h) using an Ettan DALT*six* Large Vertical System (GE Healthcare). At least three biological replicates were analyzed for each condition.

The protein spots were visualized with Coomassie Brilliant Blue G-250 (Bio-Rad Laboratories Inc.) staining (blue silver staining) according to Candiano et al.[33]. The 2-DE maps were digitalized using an Image Scanner III LabScan 6.0 (GE Healthcare). Volume spot quantitation and normalization were performed with Prodigy SameSpot software (Nonlinear Dynamics; Newcastle, UK). The volume of each spot was calculated and normalized by referring the values to the sum of total spot volumes within each gel. Student’s *t* test for unpaired samples was applied. A protein was considered differentially expressed if the mean normalized spot volume varied at least 1.4-fold between compared spots. The effect was confirmed by analysis of variance at a significance level of *p*<0.05. Protein spots showing significant variation between the studied conditions were manually excised from the gels and analyzed by Maldi ToF—MS-MS peptide mass fingerprinting (PMF).

Selected protein spots were destained and digested overnight at 37°C with 12 ng/mL proteomic grade trypsin (Promega). Peptides were subsequently ionized using α-cyano-4-hydroxycinnamic acid (10 mg/mL in 50% acetonitrile/0.1% TFA) as the matrix. Mass spectra were obtained on a MALDI-TOF Voyager-DE^TM^ PRO mass spectrometer (Applied Biosystems), operating in the reflectron positive ion mode. The laser intensity (N_2_, 337 ns) was just above the threshold of ion generation. Mass spectra were acquired from each sample by accumulating 200 laser shots. A peptide mixture (Sigma-Aldrich Co.) was used as external standard. Mass spectrometric analysis was performed using an ABI 4700 Proteomics Analyzer (Applied Biosystems) Maldi ToF—MS-MS spectrometer. Proteins were identified using Mascot Software (Matrix Science Inc.; Boston, MA, USA; http://www.matrixscience.com/search_form_select.html), based on the annotated genome of *L*. *reuteri* DSM 20016 (http://www.ncbi.nlm.nih.gov/nuccore/NC_009513.1). Search results were filtered according to the following criteria: databases, NCBI and/or SwissProt; taxonomy, Bacteria (Eubacteria) or Other Firmicutes; type search, MS/MS ion search; enzyme, trypsin; fixed modifications, carbamidomethylation (C); mass values, monoisotopic; peptide mass tolerance, 1 Da; Fragment mass tolerance, 0.7 Da; max missed cleavages, 0.

### Shotgun proteomic analysis

The entire protein lysates (60 μg) from 24 h-cultures grown in CDM_G_ and CDM_GF_ were subjected to reduction/alkylation of cysteines and trypsinolysis. Briefly, cysteines were reduced in 1 mL of 6 M guanidine HCl, 300 mM Tris-HCl, 1 mM EDTA, 10 mM DTT, pH 8.0, 1 h at 56°C and then alkylated with iodoacetamide (55 mM, final concentration) 30 min at RT in the dark. Proteins were immediately desalted with PD-10 Desalting Columns (Amersham BioSciences; Little Chalfont, Buckinghamshire, UK) using 25 mM ammonium bicarbonate pH 8.0 as the eluent. Proteins were quantified using the Bradford method and proteolyzed at 37°C overnight using proteomic grade modified trypsin (Promega; Madison, WI, USA) using a 50:1 (w/w) protein-to-trypsin ratio. Peptide solutions were lyophilized and reconstituted several times with 0.1% (v/v) trifluoroacetic acid (TFA).The tryptic digests were separated by μHPLC using a BioCAD Integral 100Q HPLC system (PerSeptive Biosystems; Framingham, MA, USA). The flow rate was regulated from 200 to 5 μL/min using a flow splitter using the following eluents: A) 5% acetonitrile containing 0.08% (v/v) formic acid and 0.01% (v/v) TFA, and B) 95% acetonitrile containing 0.08% (v/v) formic acid and 0.01% (v/v) TFA. Peptides derived from protein fractions were separated on a Acclaim^®^ PepMap300 C18 column (15 cm length, 300 μm i.d., 300 Å pore size, Dionex, Sunnydale, CA, USA) using a linear gradient from 5 to 40% of solution B for 90 min. The MS/MS analysis was performed with a Q-Star Pulsar (Applied Biosystems; Foster City, CA, USA) mass spectrometer equipped with an electrospray ion source. The precursor ions were selected for MS/MS fragmentation using the following criteria: a minimum ion mass-to-charge ratio (m/z) of 400.0, +1 to +4 charges, and 50.0 mmu tolerance for precursor ions. Multi-charged ions were fragmented using collision-induced dissociation (CID) and nitrogen as collision gas.

Raw data from μHPLC-MS/MS were used to generate peak lists in mascot generic files (.mgf) for searching with Mascot (http://www.matrixscience.com) and Protein Prospector-Batch Tag (http://prospector.ucsf.edu/prospector/mshome.htm) search engines. Search results were filtered according to the following criteria: databases, NCBI; taxonomy, other Firmicutes; type search, MS/MS ion search; enzyme, trypsin; fixed modifications, carbamidomethylation (C); variable modifications, methionine oxidation, acetyl (N-term); deamidated (NQ) and the pyro-glutamic formation through the loss of N-terminal of Gln (Gln->pyro-Glu); mass values, monoisotopic tolerance, 0.08 Da; MS/MS tolerance, 0.3 Da; missed cleavages allowed, up to 1.

## Results

### Influence of fructose in the microbial kinetics and the MDH activity of *L*. *reuteri* CRL 1101

Despite a few studies done on the isolation, heterologous expression, and characterization of MDH, whether its expression is constitutive or inducible in LAB has not been investigated. To determine if the MDH activity was induced by the presence of fructose in *L*. *reuteri* CRL 1101, this microorganism was grown in MRS broth with 2% (w/v) glucose and 5% (w/v) fructose (MRS_GF_) as carbon sources at 37°C for 24 h. MRS broth with 7% (w/v) glucose (MRS_G_) was used as negative control for mannitol production. In this assay, optimum sugar concentrations (7% w/v) for mannitol production were used as reported previously [14]. The specific enzymatic activity was evaluated in intracellular protein extracts from cells harvested at 4, 8, and 24 h of incubation. In addition, cell growth (OD_600_ and CFU/mL), medium pH, mannitol production and residual sugar concentration were determined throughout the fermentation.

**Fig 2:**
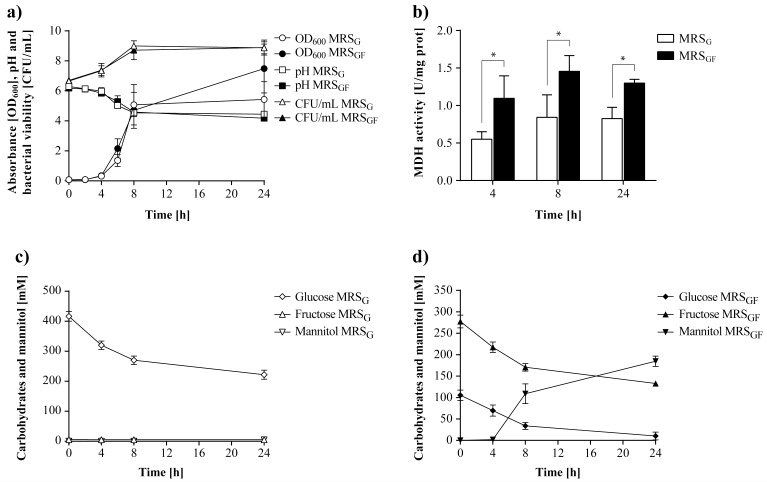
Cell growth and MDH activity of *L*. *reuteri* CRL 1101 grown in MRS_G_ and MRS_GF_ at 37°C for 24 h. a) Growth kinetics in both media; b) Specific MDH activity in both media; c, d) Carbohydrate consumption and mannitol production in MRS_G_ and MRS_GF_, respectively. Statistical analysis in Fig 2b was performed using two-way ANOVA followed by Bonferroni post-test. A value of *p<*0.05 was considered statistically significant.

Cell growth of *L*. *reuteri* CRL 1101 was similar in both culture media MRS_G_ and MRS_GF_ (Fig 2A). The highest cell count values (9.1 ± 0.1 and 8.7 ± 0.6 log CFU/mL, respectively) were obtained at 8 h of incubation. From this time point, the values remained constant until the end of the fermentation. In both media, the pH values decreased to 4.5–4.6 during the first 8 h, reaching values of 4.2–4.4 at the end of fermentation. A fast glucose uptake was observed during the first 8 h, which decreased at the beginning of the stationary phase (Fig 2C and 2D). In MRS_GF_ glucose was almost depleted after 24 h incubation, while in MRS_G_ 53% of the initial glucose (416 ± 16 mM) remained unfermented. Mannitol production was detected at 4 h of incubation in MRS_GF_; the maximum concentration obtained was 184 ± 12 mM (34 ± 2 g/L) at 24 h (Fig 2D). As expected, no mannitol was found in MRS_G_. Interestingly, a basal MDH activity value was detected at all evaluated time points in MRS_G_ (negative control) despite the culture medium did not contain fructose (Fig 2B). The highest MDH activity value (0.8 ± 0.3 U/mg prot) was observed at 8 h of incubation. MDH activity was increased in the presence of fructose (MRS_GF_) being 1.5 ± 0.2 U/mg prot the maximum value detected at 8 h, 1.9 times greater than that observed in the control.

**Fig 3:**
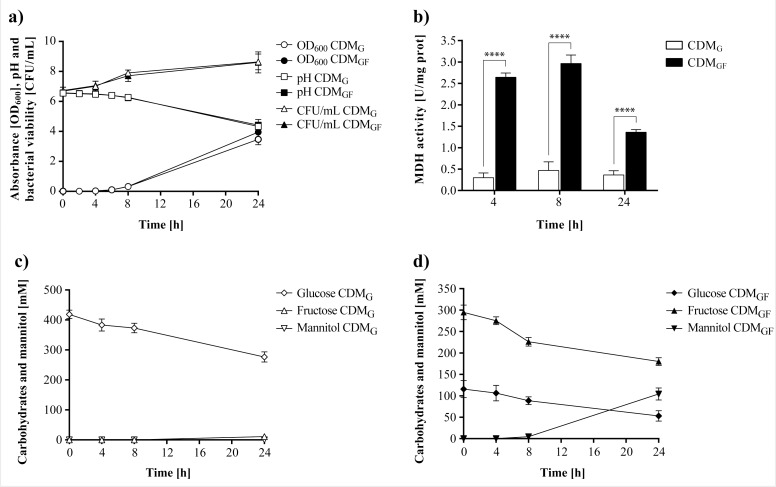
Cell growth and MDH activity of *L*. *reuteri* CRL 1101 grown in CDM_G_ and CDM_GF_ at 37°C for 24 h. a) Growth kinetics in both media; b) Specific MDH activity in both media; c, d) Carbohydrate consumption and mannitol production in CDM_G_ and CDM_GF_, respectively. Statistical analysis in Fig 3b was performed using two-way ANOVA followed by Bonferroni post-test. A value of *p<*0.05 was considered statistically significant. ns *p*>0.05, * *p*<0.05, ** *p*<0.01, *** *p*<0.001, **** *p*<0.0001.

The MRS_G_ medium contained traces of fructose (~5 mM) and sucrose (~2 mM) as determined by HPLC (S1 Fig) that might have induced the basal MDH activity observed in MRS_G_. To better evaluate whether this enzyme was constitutive or inducible by fructose, the assays were repeated using a chemically defined medium (CDM) with the same type and sugar concentrations (CDM_G_ and CDM_GF_) as before. Cell growth of *L*. *reuteri* CRL 1101 in CDM was lower than in MRS, and a maximum cell count of 8.6–8.7 log CFU/mL at the end of fermentation in both CDM was reached (Fig 3A). After 8 h of incubation, the cultures were still at the log growth phase and a scarce reduction in pH (0.29–0.33 units) was observed; the pH values decreasing 2.23–2.15 units after 24 h. Concomitantly, sugar consumption was slower in CDM_GF_ than in MRS_GF_ and no depletion of glucose was observed in CDM_GF_ (Fig 3D). In this medium, mannitol was detectable after8 h, reaching a maximum value of 104 ± 8 mM (19 ± 2 g/L) at 24 h. This value corresponds to 56% of that achieved in MRS_GF_ at the same time point. As observed in MRS_G_, a basal MDH activity value although at lesser extent was detected in CDM_G_ at all evaluated times despite the absence of fructose in the culture medium (Fig 3B). In CDM_GF_, a 9- and 6-fold increase in the MDH activity (2.6 ± 0.1 and 3.0 ± 0.2 U/mg prot) at 4 and 8 h, respectively, was observed as compared to the control (0.3 ± 0.1 and 0.5 ± 0.2 U/mg prot). While this basal activity in CDM_G_ was lower than that found in MRS_G_, the detected activity values suggested that the enzyme MDH is markedly induced by fructose.

### Expression of the *mdh* gene in the presence of fructose

**Fig 4:**
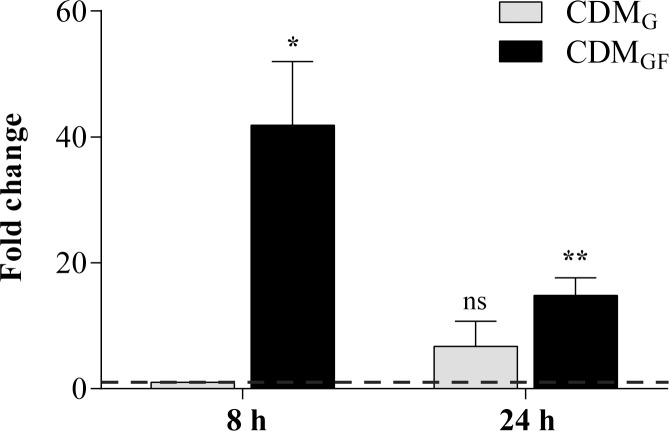
Relative expression of the *mdh* gene in *L*. *reuteri* CRL 1101 in presence (CDM_GF_) and absence (CDM_G_) of fructose incubated at 37°C after 8 and 24 h of incubation. Statistical analysis was performed using one sample *t*-test comparing all values with an hypothetical value of one (control condition = CDM_G_ 8 h). Differences between groups were considered to be significant at a *p* value of <0.05. ns *p*>0.05, * *p*<0.05, ** *p*<0.01.

The relative expression of the *mdh* gene in the absence and presence of fructose was determined by qPCR using 8 h- (log phase) and 24 h- (stationary phase) cells grown in CDM_G_ and CDM_GF_. The expression levels of four housekeeping genes (*16S rRNA*, *gyrB*, *pyrG* and *leuS*) typically used as normalizing genes, were also tested. The *pyrG* gene (coding for the enzyme CTP synthetase involved in the nucleotide metabolism) was selected as normalizing gene as its transcription level remained stable in all the assayed conditions. The 2^-ΔΔCT^ method [31] was used for the interp retation of the results while 8 h of incubation in the CDM_G_ medium was the condition used as reference to which the arbitrary value of 1 was assigned. The relative values of *mdh* gene expression in the absence and presence of fructose at 8 and 24 h of incubation are shown in Fig 4. Cycle threshold (C_T_) values obtained for both genes under all assayed conditions are shown in Table 1. A basal *mdh* gene expression was detected in the absence of fructose (CDM_G_) in both the log (8 h, control) and stationary (24 h) phases, the latter value being 6.7 ± 4.1 times greater than that obtained in the control. The *mdh* gene expression increased 42-fold (41.8 ± 10.2) in the log phase (8 h) in the presence of fructose (CDM_GF_) as compared to the control; interestingly, the over-expression of *mdh* was not so notable at 24 h being the obtained value (14.8 ± 2.8) 15-fold greater than the control. Overall, these data demonstrate that the *mdh* gene in *L*. *reuteri* CRL 1101 is markedly up-regulates in the presence of fructose, especially during the log phase.

**Table 1:** C_T_ values of *mdh* and *pyrG* genes of *L*. *reuteri* CRL 1101 grown in CDM in absence and presence of fructose (CDM_G_ and CDM_GF_) for 8 and 24 h.

Time (h)	Condition	Gene	C_T_ value	
			Average	SD
8	CDM_G_	*mdh*	23.53	0.16
		*pyrG*	21.28	0.95
	CDM_GF_	*mdh*	17.01	0.28
		*pyrG*	20.13	0.47
24	CDM_G_	*mdh*	20.69	1.25
		*pyrG*	21.23	2.16
	CDM_GF_	*mdh*	19.61	1.40
		*pyrG*	21.42	0.17

SD: standard deviation.

### Proteomic analysis

#### Differential expression of intracellular proteins of *L*. *reuteri* CRL 1101 during mannitol production by 2DE analysis

As several changes occur in microbial metabolism in the presence of different carbon sources [34–36], a global proteome response of *L*. *reuteri* CRL 1101 to the presence of fructose was evaluated by 2DE and shotgun proteomics.

**Fig 5:**
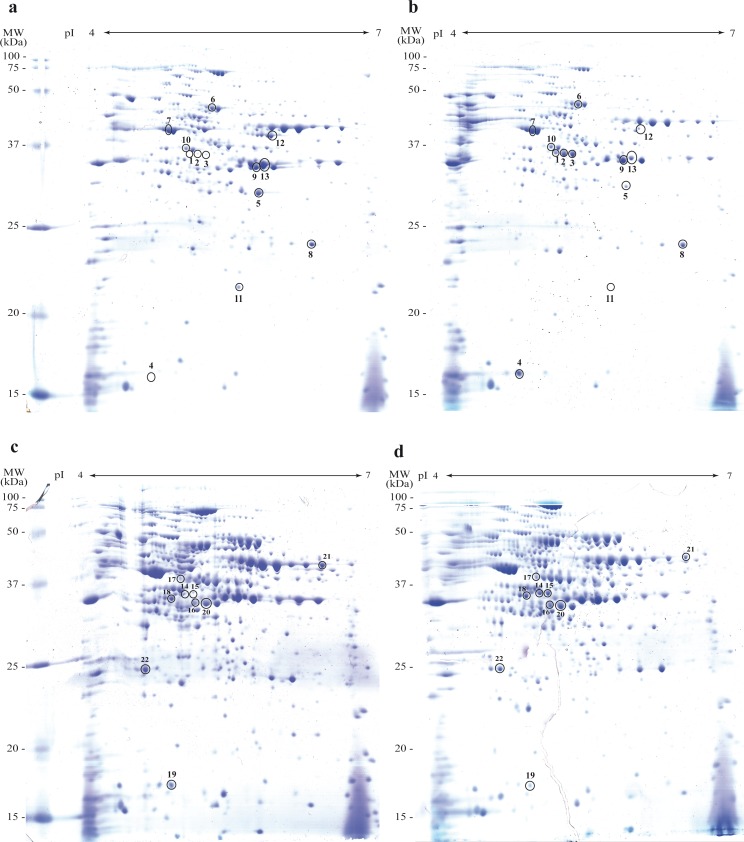
Representative gel images of proteomes of *L*. *reuteri* CRL 1101 obtained under the four studied conditions. a, b) 8 h of incubation in the absence (control) and in the presence of fructose, respectively; c, d) 24 h of incubation in the absence and in the presence of fructose, respectively. Circles indicate the identified spots by MALDI-TOF. Linear pH gradient was used.

**Fig 6:**
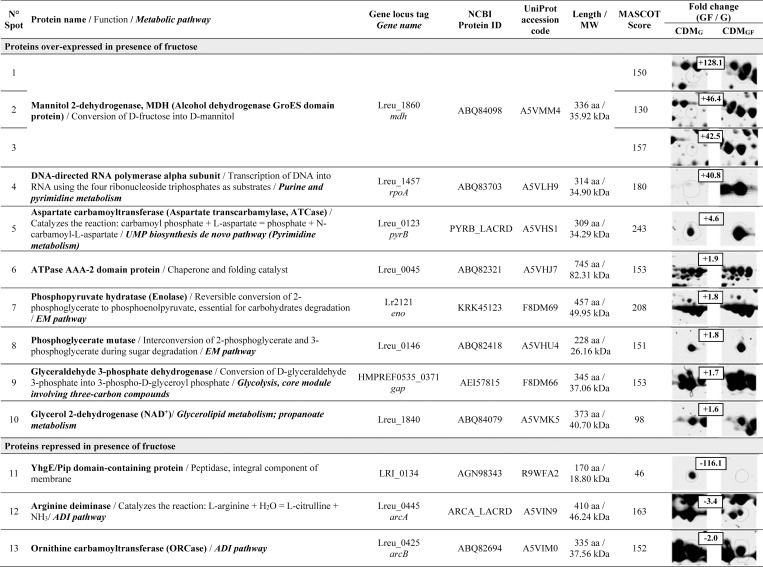
Proteins of *Lactobacillus reuteri* CRL 1101 over-expressed or repressed at 8 h of incubation in the presence of fructose, separated by 2DE and identified by Maldi ToF–MS-MS.

**Fig 7:**
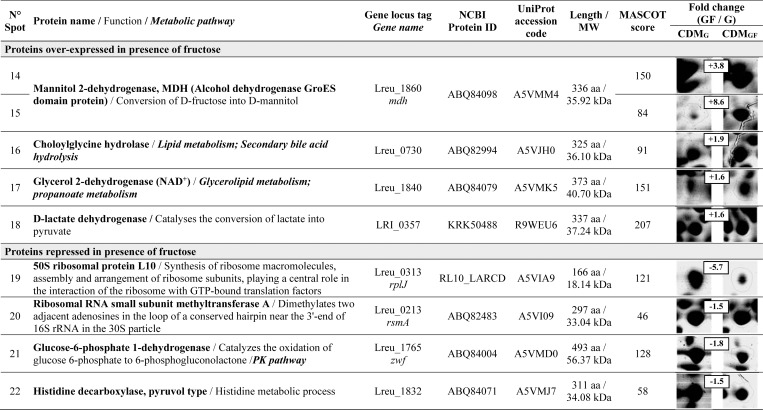
Proteins of *Lactobacillus reuteri* CRL 1101 over-expressed or repressed at 24 h of incubation in the presence of fructose, separated by 2DE and identified by MS.

The 2DE maps of exponential (8 h) and stationary (24 h) cells growing in the presence (CDM_GF_, mannitol production condition) and in the absence of fructose (CDM_G_, no mannitol production, negative control) allowed the detection, using software-assisted image analysis, of approximately 300 spots in each condition, from which 30 spots, showing significant differential expression (*p<*0.05),were selected for identification by MS. Representative 2DE maps in the 4–7 pI range of the entire bacterial lysates from 8- and 24-h cultures are depicted in Fig 5. Twenty-two spot proteins were differentially expressed and successfully identified by MS (these proteins appear labeled with circles in Fig 5). The differential expression levels ranged from 1.5 to 128.1 fold variations (*p*<0.05). The MS qualitative results of the identified spots, putative assigned functions, and relative quantification of differential expression of proteins done by densitometry are shown in Figs 6 and 7. In the presence of fructose at 8 h of incubation, 13 proteins were differentially expressed, 10 of which were over-expressed and 3 were negatively regulated (Fig 6); whereas at 24 h of incubation, 9 proteins changed their expression profiles in the presence of fructose: 4 were up-regulated and 5 were down-regulated (Fig 7).

**Fig 8:**
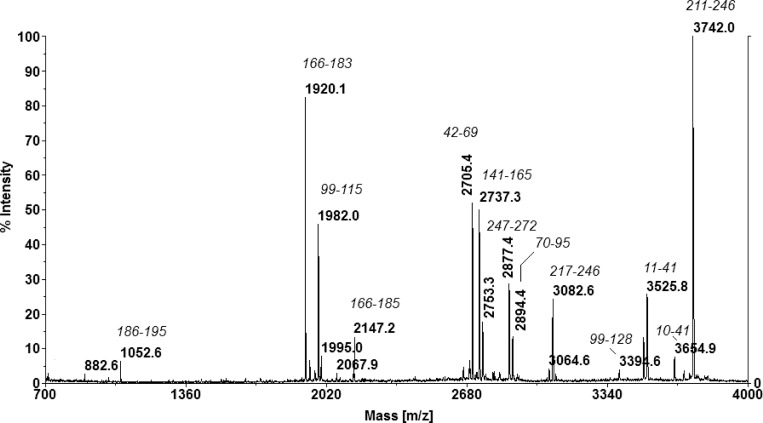
Maldi ToF—MS-MS mapping of MDH. Signals are assigned through the position within the protein as presented in italics.

Among the proteins differentially expressed at 8 h of incubation in the presence of fructose, the MDH enzyme was clearly over-expressed (spot 1, 128-fold increase; spot 2, 46-fold increase; spot 3, 42-fold increase). In contrast, these spots were almost missing in the gels of cells grown in the absence of fructose. The Maldi ToF—MS-MS mapping of MDH is shown in Fig 8. The occurrence of MDH in three spots differing by approximately 0.1 pI units could be attributed to post-translational modifications that we were not able to assign with our analysis. Most likely, these discrepancies reflect a variable degree of protein phosphorylation. On the other hand, 2 proteins related to purine and pyrimidine metabolism were positively regulated in this condition: DNA-directed RNA polymerase alpha subunit (spot 4, 40.8-fold increase) and aspartate transcarbamylase (spot 5, 4.6-fold increase). Moreover, 3 proteins related to carbohydrate degradation through the Embden-Meyerhof-Parnas (EMP) pathway were 1.7- and 1.8-fold over-expressed: enolase, phosphoglycerate mutase, and glyceraldehyde 3-phosphate dehydrogenase (spots 7, 8, and 9, respectively). It is noteworthy that 2 out of the 3 proteins belonging to the ADI (Arginine Deiminase) pathway were repressed: ADI (spot 12; 3.4-fold decrease) and ornithine carbamoyltransferase (spot 13; 2-fold decrease). In this condition, YhgE/Pip domain-containing protein, an integral component of the plasma membrane, was clearly down-regulated (spot 11; 116-fold decrease).

After 24 h of incubation in the presence of fructose, the MDH enzyme was up-regulated (spot 14, 3.8-fold increase; spot 15, 8.6-fold increase) although at lesser extent than at 8 h incubation. In this condition, other 3 proteins showed an increased expression: choloylglycine hydrolase (spot 16, 1.9-fold), glycerol 2-dehydrogenase (spot 17, 1.6-fold), both enzymes involved in the lipid metabolism, and D-lactate dehydrogenase (spot 18, 1.6-fold) involved in the conversion of lactate into pyruvate through the EMP pathway. Concerning the down-regulated proteins, i) the 50S ribosomal protein L10 (spot 19, 5.7-fold)involved in the synthesis of ribosome macromolecules and assembly and arrangements of ribosome subunits; ii) the ribosomal RNA small subunit methyltransferase A (spot 20, 1.5-fold), which may play a critical role in biogenesis of 30S subunits; iii) the glucose 6-phosphate 1-dehydrogenase (spot 21, 1.8-fold) involved in the phosphoketolase pathway, and finally iv) the histidine decarboxylase (spot 22, 1.5-fold) involved in histidine metabolic processes, were under-expressed when *L*. *reuteri* CRL 1101 grew in the presence of fructose after 24 h of incubation.

### Shotgun proteomic analysis of whole lysates of *L*. *reuteri* CRL 1101 grown in the presence of fructose

To obtain a more complete coverage of the proteome of *L*. *reuteri* in response to the presence of fructose, we performed shotgun proteomics of cells grown during 24 h in the presence and in the absence of fructose. In fact, only 8 and 25 gene products were identified in the CDM_G_ and CDM_GF_ grown cells, respectively (Table 2). However, MDH was clearly identified with 29.7% coverage (protein N° 21, 6 unique peptides matching) in the protein extracts from cells grown in CDM_GF_, which was not detected in the counterpart grown in CDM_G_. Three proteins have been detected both in the absence and in the presence of fructose: phosphoketolase (proteins N° 1 and 33), elongation factor Tu (proteins N° 2 and 10), and phosphopyruvate hydratase (proteins N° 3 and 14). It is worth noting that 5 proteins have been identified through both techniques, 2DE and shotgun: phosphopyruvate hydratase (spot 7; shotgun proteins N° 3 and 15), glyceraldehyde 3-phosphate dehydrogenase (spot 9; shotgun protein N° 4), phosphoglyceromutase (spot 8; shotgun protein N° 19), mannitol dehydrogenase (spots 1, 2, 3, 14, and 15; shotgun protein N° 21), and D-lactate dehydrogenase (spot 18, shotgun protein N° 26).

**Table 2:** Shotgun proteomic identification of proteins of *L*. *reuteri* CRL 1101 whole lysates, grown in the absence and in the presence of fructose for 24 h. Score and mass values are reported along with the number of identified peptides (matches) and gene locus tag. In the current experimental conditions, matches with score values >30 were considered proof of identity or extensive homology (*p*<0.01).

N° Protein	Accession number	Score	Mass	Matches	Gene locus tag	Description
**In the absence of fructose (CDM**_**G**_**)**						
1	gi|148531926	179	91403	14	Lreu_1686	Phosphoketolase [*Lactobacillus reuteri*]
2	gi|489762899	156	43405	4	Lreu_0651	Elongation factor Tu OS = *Lactobacillus reuteri* (strain DSM 20016)
3	gi|489762504	148	48010	11	lr2121	Phosphopyruvate hydratase [*Lactobacillus reuteri*]
4	gi|227071557	108	37267	7	HMPREF0535_0371	Glyceraldehyde-3-phosphate dehydrogenase, type I [*Lactobacillus reuteri*MM2-3]
5	PGK_LACRJ	52	42934	2	LAR_0382	Phosphoglycerate kinase OS = *Lactobacillus reuteri* (strain JCM 1112)
6	Y1202_LACRJ	43	14087	1	LAR_1202	UPF0342 protein LAR_1202 OS = *Lactobacillus reuteri* (strain JCM 1112)
7	RS8_LACRD	35	14527	3	Lreu_1469	30S ribosomal protein S8 OS = *Lactobacillus reuteri* (strain DSM 20016)
8	RS5_LACRD	31	17636	2	Lreu_1466	30S ribosomal protein S5 OS = *Lactobacillus reuteri* (strain DSM 20016)
**In the presence of fructose (CDM**_**GF**_**)**						
9	gi|489762899	160	43405	12	Lreu_0651	Elongation factor Tu [*Lactobacillus reuteri*]
10	gi|227070649	83	44438	1	HMPREF0535_1267	Alcohol dehydrogenase, iron-dependent [*Lactobacillus reuteri* MM2-3]
11	gi|489765780	82	60442	4	Lreu_0131	Formate-tetrahydrofolate ligase [*Lactobacillus reuteri*]
12	gi|489763202	73	50027	1	Lreu_0176	Pyridinenucleotide-disulfideoxidoreductase [*Lactobacillus reuteri*]
13	gi|489760587	72	13678	1	HQ33_09245	30S ribosomal protein S13 [*Lactobacillus reuteri*]
14	gi|489762504	66	48010	10	lr2121	Phosphopyruvate hydratase [*Lactobacillus reuteri*]
15	gi|489761501	65	57452	6	Lreu_0144	Bifunctionalphosphoribosylaminoimidazolecarboxamideformyltransferase/Inosine monophosphate cyclohydrolase [*Lactobacillus reuteri*]
16	gi|489760571	63	16294	1	Lreu_1451	50S ribosomal protein L13 [*Lactobacillus reuteri*]
17	gi|489761434	62	32655	1	Lreu_0102	Ribonucleosidehydrolase RihC [*Lactobacillus reuteri*]
18	gi|227071420	62	26136	1	Lreu_0164	Putative dihydrodipicolinate reductase domain protein [*Lactobacillus reuteri* MM2-3]
19	gi|489761508	60	26162	1	Lreu_0146	Phosphoglyceromutase [*Lactobacillus reuteri*]
20	gi|227070837	58	56679	2	Lreu_1765	Glucose-6-phosphate dehydrogenase [*Lactobacillus reuteri* MM2-3]
21	gi|45268465	55	36284	6	Lreu_1860	Mannitol dehydrogenase [*Lactobacillus reuteri* ATCC 53608]
22	gi|489764864	54	36615	5	Lreu_1496	Zinc-dependent alcohol dehydrogenase [*Lactobacillus reuteri*]
23	gi|148531697	52	14243	1	Lreu_1450	SSU ribosomal protein S9P [*Lactobacillus reuteri* DSM 20016]
24	gi|194454156	49	37028	1	LRI_0357	D-lactate dehydrogenase [*Lactobacillus reuteri* I5007]
25	gi|93280020	49	34627	1	Lreu_1206	Glucokinase [*Lactobacillus reuteri*]
26	gi|489761486	48	49691	4	Lreu_0136	Adenylosuccinatelyase [*Lactobacillus reuteri*]
27	gi|489760284	45	64763	1	Lreu_1313	Phosphoenolpyruvate carboxykinase [*Lactobacillus reuteri*]
28	gi|146345335	44	31965	1	Lreu_1285	Sugarkinase [*Lactobacillus reuteri*]
29	gi|68161005	43	31007	1	Lreu_0354	Chaperonin GroEL [*Lactobacillus reuteri* DSM 20016]
30	gi|489761003	43	53498	8	Lreu_1766	6-phosphogluconate dehydrogenase [*Lactobacillus reuteri* DSM 20016]
31	gi|489761367	36	15827	1	Lreu_0042	OsmC family protein [*Lactobacillus reuteri* DSM 20016]
32	gi|227070122	35	34936	2	Lreu_0716	Malate dehydrogenase (NAD) [*Lactobacillus reuteri* DSM 20016]
33	gi|489760969	33	91374	8	Lreu_1686	Phosphoketolase [*Lactobacillus reuteri*]

## Discussion

To date purification and characterization of the MDH enzyme as well as the heterologous expression of the *mdh* gene have been conducted in heterofermentative LAB species such as *L*. *brevis* [16], *Leuc*. *mesenteroides* [29], *Leuc*. *pseudomesenteroides* [18], *L*. *reuteri* [28], *L*. *intermedius* [27], and *L*. *sanfranciscensis* [26]. In this work, a detailed assessment of the MDH activity and relative expression of the *mdh* gene during the growth and mannitol production by *L*. *reuteri* CRL 1101 in the presence and absence of fructose at different incubation times is presented. We showed that the MDH enzyme of *L*. *reuteri* CRL 1101 is highly induced by fructose during the early stages of microbial growth, showing a 6-fold higher activity value (3.0 ± 0.2 U/mg prot) compared to thecontrol (absence of fructose) at 8 h of incubation using a CDM. The applied synthetic culture medium was appropriate for conducting enzyme activity and gene expression studies as the exact composition of the medium was known; thus, the presence of inducers or inhibitors of the enzyme or gene of interest was considered. Previous studies on the MDH activity from *L*. *reuteri* CRL 1101 under different culture conditions (agitated and static cultures, and lower incubation temperatures than 37°C) were reported by our group. The highest activity values (3.646 U/mg protein) were found during early microbial growth phases (log phase), independently of the assayed culture conditions [14]. Additionally, when the strain was grown at constant pH in a range between 6.0 and 4.8, no significant differences in the obtained MDH activity values were observed [15]. Here, the enzyme activity values of *L*. *reuteri* CRL 1101 grown in CDM were higher than those obtained with other heterofermentative LAB strains such as *L*. *intermedius* NRRL B-3693 (0.99 U/mg prot) [27], *L*. *sanfranciscensis*TMW1.392 (1.0 U/mg prot) [26] or *Leuc*. *mesenteroides* (0.68 U/mg prot) [29]. Conversely, Hahn et al. [18] reported the highest MDH activity value (15.0 U/mg prot) for the strain *Leuc*. *pseudomesenteroides* ATCC 12291.

The quantification of the *mdh* mRNA levels of *L*. *reuteri* CRL 1101 during mannitol production *vs* the non-production condition by qPCR was performed. The expression of the constitutive *mdh* gene of this strain was markedly up-regulated when fructose was present; a 40-fold increase during the log phase as compared to the control was observed. The direct correlation between the high levels of *mdh* gene expression and the maximum MDH enzyme activity in the log growth phase suggest that the regulation of the gene expression occurs during transcription (mRNA synthesis).To the best of our knowledge, this is the first study on the expression of *mdh* mRNA in LAB. Only a few reports have shown *mdh* transcriptional studies in other bacteria. Recently, Groisillier et al. [37] evaluated the expression of genes involved in mannitol catabolism by the marine heterotrophic bacterium *Zobellia galactanivorans*. When this bacterium was cultured in the presence of mannitol as the sole carbon source, five genes coding for the proteins MDH and fructokinase, and an ATP binding cassette (ABC) transporter complex were induced suggesting the organization into one operon. In another study, the expression of both enzymes NADH- and NADPH-dependent MDH has been evaluated in the bacterium *Gluconobacter oxydans* growing under osmotic stress conditions in the presence of sucrose [38]. NADPH-dependent MDH was determinant for mannitol production as the mRNA abundance of this enzyme was 30-fold higher than the NADH-dependent enzyme. Accordingly, the NADH-dependent MDH activity was 10-fold lower than the NADPH-dependent MDH. Also, the *mdh* gene expression was studied in the brown algae *Saccharina japonica* under osmotic, oxidative, and desiccative stress conditions. The mRNA levels of the *mdh* (*SjM2DH*) were analyzed under different NaCl (400–1200 mM) concentrations. The higher transcription level was obtained with 400 mM of NaCl, which decreased with the increase in NaCl concentration. On the other hand, a remarkable up-regulation of *SjM2DH* was found under 0.8 mM H_2_O_2_, which was 60-fold higher than that at 0.2 mM. Moreover, its mRNA level was 7-fold higher after 2 h of desiccation. These results suggest that mannitol might have a role in osmotolerance and could be involved in the desiccative and oxidative stress response in *S*. *japonica* [39].To date, studies on genes involved in mannitol metabolism have been reported mainly in fungi. In *Alternaria brassicicola*, the relative expression of the *mpd* gene, encoding for the mannitol 1-P dehydrogenase enzyme (MPD, which catalyzes the conversion of fructose-6-P into mannitol-1-P) and the *mdh* gene after 24 h-exposure to plant defense compounds such as isothiocyanates (ITC) was determined [40]. As the relative *mpd* gene expression remained unchanged while the *mdh* gene expression increased 3 times, it was proposed that mannitol participates in fungal protection against oxidative stress generated by ITC exposure.

Heterofermentative LAB use the 6-phosphogluconate/phosphoketolase (PK) pathway for carbohydrate degradation. When these bacteria grow on glucose plus fructose, they preferentially use glucose as carbon source for cell metabolism producing equimolar amounts of lactic acid, ethanol, and carbon dioxide. Fructose instead, may be used as an external electron acceptor being reduced to mannitol; this reaction contributes to the replenishment of the cells’ NAD(P)^+^ pool. In these conditions, the cells switch to produce acetate and one extra ATP molecule rather than ethanol [3,41]. When unraveling the functions involved in response to the presence of fructose in *L*. *reuteri* CRL 1101 by applying proteomics, the MDH and D-lactate dehydrogenase enzymes were, among other proteins, up-regulated in the presence of fructose. Growth of *L*. *reuteri* CRL 1101 on glucose as the sole carbon source (CDM_G_) resulted in an increased ethanol formation. The presence of an alternative external electron acceptor such as fructose allowed mannitol formation, NAD^+^ regeneration, restoring consequently the redox equilibrium, and acetate synthesis. By this means, ATP generation and higher biomass production are achieved, being this favorable energy cell state the real reason for mannitol production [42]. In addition, other proteins involved in i) carbohydrate catabolism; ii) purine and pyrimidine metabolism, and iii) in a lesser extent, lipid metabolism were up-regulated. Part of these observations were confirmed by other authors who compared the transcriptomes of the strains *L*. *reuteri* ATCC 55730 and ATCC 6475 showing that both the EMP and PK pathways are active during the exponential and stationary growth phases [43]. Concerning the lipid metabolism, the enzyme glycerol 2-dehydrogenase (Lreu_1840) involved in glycerol formation was over-expressed in the 8 h- (spot 10; 1.6-fold increase,Fig 6) and 24 h-cultures (spot 17; 1.6-fold increase,Fig 7) of *L*. *reuteri* CRL 1101in the presence of fructose. Accordingly, when Bustos et al. [44] studied the molecular mechanisms involved in the adaptation of the probiotic strain *L*. *reuteri* CRL1098 to bile acids by a proteomic approach, the isoenzyme Lreu_1840 responsible for glycerol production was over-expressed in the presence of both conjugated- and free- bile acids. Interestingly, the enzymes arginine deiminase and ornithine carbamoyl transferase, involved in the arginine metabolism through the ADI pathway were down-regulated in the presence of fructose. As the ADI pathway allows energy generation under stress culture conditions [45], our findings hint at the possibility that in the presence of fructose no need of ATP generation through the ADI pathway is required since acetate formation with the concomitant production of extra ATP occur when mannitol is produced by *L*. *reuteri* CRL 1101.

Both proteomic approaches (2DE and shotgun) applied in this study confirm the inducible expression of MDH by *L*. *reuteri* at either the exponential or the stationary phases in the presence of fructose. It could be expected that the activation of the MDH synthesis is associated with a metabolic re-arrangement of the regulatory networks. The medium-induced shift of the metabolic pathways is a clear example of metabolic engineering, intended as the control and the optimization of the enzymatic and regulatory pathways to induce or increase the production of a specific metabolite.

A global analysis on the MDH activity and its differential protein and mRNA expression by the presence of fructose is for the first time reported in a lactic acid bacterium strain. *L*. *reuteri* is one of the LAB species most frequently used as a probiotic agent in humans and animals [46–48], being one of the few endogenous *Lactobacillus* species found in the gastrointestinal tract of several mammals [49,50]. In addition, *L*. *reuteri* has been found in the natural microbiota of sourdoughs of different cereals and of the pseudocereal buckwheat [51,52]. This species, which can dominate sourdoughs subjected to high temperatures and prolonged fermentation times, has been shown to persist in industrial sourdough for up to ten years [53,54]. Despite being naturally present in this ecosystem and being able to grow well on different substrates, cereal fermentation using *L*. *reuteri* displays beneficial effects on the nutritional value of the final product by increasing the free-phenolic acid content [55] and by the formation of γ-aminobutyric acid [52, 56]. Although the *in situ* mannitol formation by *L*. *reuteri* in fermented foods remains to be applied, our findings could be useful for the elaboration of naturally produced low-calorie fermented foods. While mannitol production seems to be widespread in the *L*. *reuteri* species, the production levels and yields of this polyol are strain-dependent [57, 58]. Furthermore, this comprehensive study on mannitol formation by *L*. *reuteri* CRL 1101 represents a deeper insight into the carbohydrate metabolism and polyol formation by a heterofermentative *Lactobacillus* strain species with biotechnological potential in the nutraceutics and pharmaceutical areas.

## Supporting Information

S1 Fig HPLC Chromatogram of sterile MRS broth (2% w/v of glucose) reveals the presence of fructose and sucrose traces. (TIF)
