# Supplementary figures and images for: Global Analysis of Mannitol 2-Dehydrogenase in Lactobacillus reuteri CRL 1101 during Mannitol Production through Enzymatic, Genetic and Proteomic Approaches
Source: PLoS One. 2017 Jan 6;12(1):e0169441. doi: 10.1371/journal.pone.0169441 (PMC5218481; doi:10.1371/journal.pone.0169441)

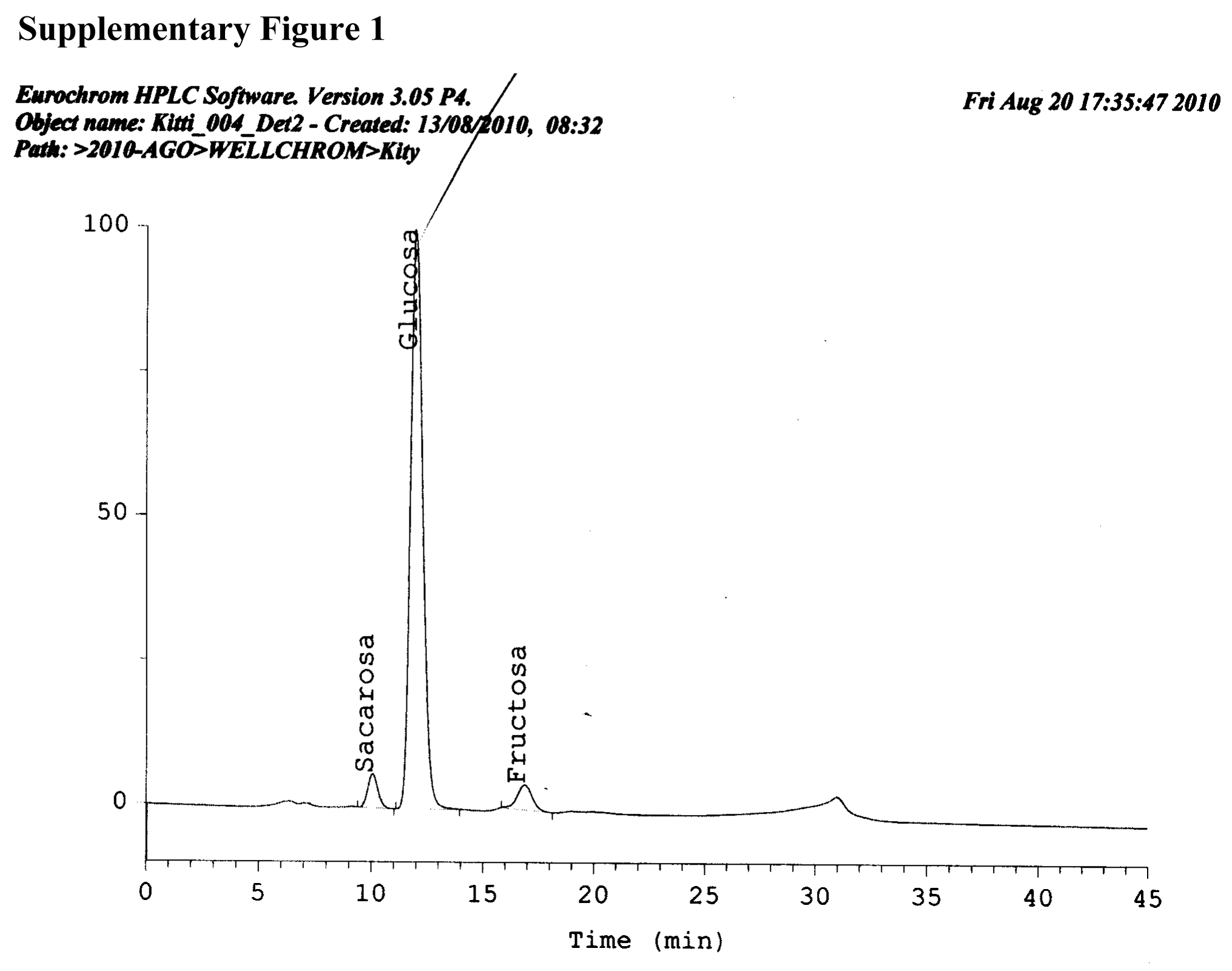

Supplement: S1 Fig — (TIF) [file pone.0169441.s001.tif]
